# A genome alignment algorithm based on compression

**DOI:** 10.1186/1471-2105-11-599

**Published:** 2010-12-16

**Authors:** Minh Duc Cao, Trevor I Dix, Lloyd Allison

**Affiliations:** 1 Clayton School of Information Technology, Monash University, Clayton 3800, Australia

## Abstract

**Background:**

Traditional genome alignment methods consider sequence alignment as a variation of the string edit distance problem, and perform alignment by matching characters of the two sequences. They are often computationally expensive and unable to deal with low information regions. Furthermore, they lack a well-principled objective function to measure the performance of sets of parameters. Since genomic sequences carry genetic information, this article proposes that the information content of each nucleotide in a position should be considered in sequence alignment. An information-theoretic approach for pairwise genome local alignment, namely XMAligner, is presented. Instead of comparing sequences at the character level, XMAligner considers a pair of nucleotides from two sequences to be related if their mutual information in context is significant. The information content of nucleotides in sequences is measured by a lossless compression technique.

**Results:**

Experiments on both simulated data and real data show that XMAligner is superior to conventional methods especially on distantly related sequences and statistically biased data. XMAligner can align sequences of eukaryote genome size with only a modest hardware requirement. Importantly, the method has an objective function which can obviate the need to choose parameter values for high quality alignment. The alignment results from XMAligner can be integrated into a visualisation tool for viewing purpose.

**Conclusions:**

The information-theoretic approach for sequence alignment is shown to overcome the mentioned problems of conventional character matching alignment methods. The article shows that, as genomic sequences are meant to carry information, considering the information content of nucleotides is helpful for genomic sequence alignment.

**Availability:**

Downloadable binaries, documentation and data can be found at ftp://ftp.infotech.monash.edu.au/software/DNAcompress-XM/XMAligner/.

## Background

Advances in sequencing technology allow high throughput production of biological sequences in sequencing laboratories around the world. The exponential increase of genomic data extracted recently introduces the need for analysis techniques that can handle the large amount of data. This is very challenging as conventional analysis methods can be overwhelmed by volume and misled by statistical biases. It is important to develop novel tools that are time efficient and able to cope with the diversity of the data.

One of the most important tools for sequence analysis, if not the most important one, is sequence alignment which attempts to arrange biological sequences to identify regions of similarity. Similarities between sequences can provide clues to discover the evolutionary relationship between species, to annotate new sequences and to compare an un-known sequence against existing sequences in a large database. There are two broad kinds of sequence alignment, namely *global alignment *and *local alignment*. Global alignment attempts to match entire sequences from end to end and thus is suitable for comparing short sequences that are expected to have similar structures and functions such as proteins or genes. On the other hand, local alignment searches for conserved regions, possibly *reordered*, between two sequences. Local alignment is thus more suitable for analysing long sequences, such as chromosomes or genomes, especially from distantly related species where significant insertions, deletions and large rearrangements may have occurred.

Most existing alignment methods are inspired by the dynamic programming approach [1,2] which attempts to examine all possible pairings of the two sequences and chooses the highest matching score alignment. This dynamic programming alignment approach has quadratic time and space complexities and hence is unattractive for handling long sequences and high volume sequence databases. To trade sensitivity for running time, heuristic search methods are often used. Instead of comparing every single base of the two sequences, FASTA [3] and BLAST [4], the two most popular database search tools, first search for *seeds *of *k *consecutive exact matches. Seeds are then extended, by limited dynamic programming, to allow for mutations and gaps.

Since 1995 when the first genome of a free-living organism was sequenced [5], a number of alignment tools capable of comparing genomes have been developed. Such examples are Gapped BLAST [6], Sim4 [7], SSAHA [8], Dialign [9], MGA [10], MUM-mer [11,12], Blastz [13], Chaos [14], and AVID [15]. Most of these methods rely on the ideas of FASTA and BLAST; they use different techniques for finding seeds and for extending seeds to identify conserved regions. Often, seeds are located by an indexing method such as hash tables (allowing or not allowing gaps), suffix trees or suffix arrays. Seeds are then extended in a fashion similar to the dynamic programming approach to form larger similar regions. Many tools chain together sufficiently near seeds, and report statistically significant chains as homologues. A comprehensive review of genome wide alignment tools is presented in [16].

Most traditional alignment methods rely heavily on a scoring scheme that includes a substitution matrix, which describes the mutation rates between nucleotides or amino acids, and other parameters such as gap penalties. However, these methods lack a well-principled objective function to measure the performance of a set of parameters: "There is considerable disagreement among biologists about the 'right' choice of parameters" [17]. Using a generic substitution matrix may be suitable for protein alignment as the rates of substitution in protein largely depend on the similarities between amino acid properties which are well understood. However, this is not the case in nucleotides; more than one codon can code for an amino acid and different strains show different codon preferences for a given amino acid [18]. It is therefore sometimes very hard to find a suitable scoring scheme for alignment of genomes, especially when little is known about the sequences. The selection of a scoring scheme would be managed easily with a reasonable objective function.

Existing alignment algorithms consider sequence alignment as a variation of the edit distance problem, and perform alignment by matching characters of the two sequences. As a result, they are unable to deal with regions of low information content such as repetitive and statistically biased DNA. Such regions are often "masked out" before alignment [19,20]. Since genomic sequences are meant to convey genetic *information*, a new alignment methodology that performs alignment based on the *information content *at each nucleotide position is proposed here. The methodology is based on information theory [21] and the *Minimum Message Length *(MML) principle [22,23]. This approach considers regions that convey similar information as potential homologues. The similarity of regions can be measured by their mutual information content.

A number of information theoretic methods have been developed to compare biological sequences. The MML encoding method [24] postulates that two sequences are related if compressing the two together results in a shorter code than the total code of compressing them separately. An extension of this information theoretic approach to alignment is Modelling-Alignment (M-Align) [25] which incorporates *population models *into the alignment process and can thus estimate the information content of each nucleotide or amino acid in context, and can change matching, insertion and deletion scores accordingly. The method has been shown to significantly reduce false positives without introducing false negatives when applied to statistically biased data. However, the quadratic complexity of M-Align prohibits applying it to long sequences.

This article presents *XMAligner*, a novel method for genomic local alignment based on information theory. As in [25,26], our work is based on the premise that if two sequences are related, one sequence must tell something useful about the other: A predictive model can predict a sequence better if a related sequence is known. The information content of a sequence is measured by lossless compression. XMAligner makes use of the expert model compression algorithm [27,28] for calculating the information content and mutual information content of the two sequences to be aligned. It does not require masking out of repetitive and low information regions. It has an objective function to help in selecting parameters for a good alignment. The method is shown to be practical and can handle sequences of eukaryote genome size.

## Method

Information theory [21] directly relates entropy to the transmission of a sequence under a compression model. Suppose a sequence *X *is to be transmitted over a reliable channel where the objective is to minimise the transmitted message. The sender compresses *X *using a compression model and transmits the encoded message to the receiver, which decodes the compressed stream, using the same model, to recover the original message. The compression is performed by the best possible compression model. The amount of information contained in *X*, or the *information content *ℐ(*X*) of *X*, is the amount of information actually transmitted across the channel, that is the length of the *compressed *message.

**Figure 1:**
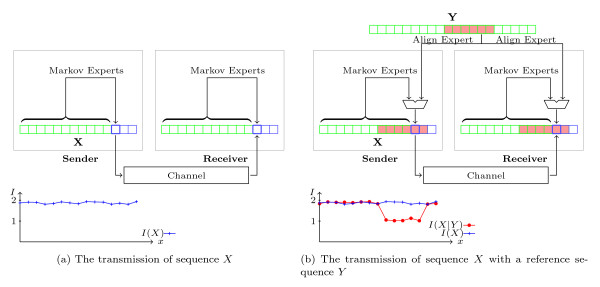
**The transmission of sequence *X *with a reference sequence *Y***. In Figure 1a, the transmission of a sequence *X *is alleviated by a compression model which uses a predictive model based on Markov experts (Markov models). The information content of each position is plotted in the graph below. With the presence of a related reference sequence *Y *, the compression model makes use of align experts, which exploit the similarity of the two sequences, for better compression (as shown in Figure 1b).

The transmission of *X *is illustrated in Figure 1a. The sender uses a predictive model, which compresses each symbol of *X *by estimating the probability of the symbol based on observation of the preceding symbols; a good prediction results in a short code-word for the symbol. The information content of every symbol makes up the *information sequence *of *X*, which is shown in the plot below the diagram.

Suppose a reference sequence *Y related *to *X *is available to both parties. The sender can further reduce the transmitted message length by transmitting only the information in *X *that is not contained in *Y *with the addition of references to the shared information contained in *Y*. The receiver can recover *X *correctly because it also knows *Y*. Since the sender aims to send the shortest possible, recoverable message, the amount of information transmitted in this case should be no more, and probably less, than the amount of information transmitted without the reference sequence The amount of information transmitted in the presence of the reference sequence *Y *is called the *conditional information content *of *X *given *Y *, denoted ℐ(*X*|*Y *). The sender is said to perform *compression of X on the background of Y*. The reduction in compressed message length caused by the presence of the reference sequence is due to the shared information between the two sequences, and hence indicates the amount of *mutual information *of the two sequences. The mutual information of *X *and *Y *is denoted as ℐ(*X*; *Y *) = ℐ(*X*)**-**ℐ(*X*|*Y *).

The transmission in the example above, but with a reference sequence, *Y *, is illustrated in Figure 1b. The predictive compression model now combines the information from all preceding symbols of *X *with the information from *Y *to estimate the probability of each symbol of *X*. If *X *and *Y *are truly related, the conditional information content of each symbol in *X*, given *Y *, will, on average, be lower than its information content without *Y*. The plot in the figure shows the sequence of information content of *X*, and the sequence of conditional information content of *X *given the reference sequence *Y*. One can notice a region in *X *that has a related region in *Y *- showing significantly lower conditional information content given *Y*.

A local alignment of two sequences shows the mapping of similar regions in the two sequences and hence reveals the references to shared information contained in the sequences. The local alignment thus allows a reduction in transmission of a sequence in the presence of the other sequence as the reference sequence. This observation leads to the proposition that optimal alignment of two sequences leads to the best compression of one sequence on the background of the other. An alignment algorithm is developed based on the proposition. It uses a compression model, which makes use of a local alignment, to compress a sequence on the background of a reference sequence, and suggests the alignment that gives the best compression. The quality of an alignment can be measured by the compression.

### The expert model

The alignment algorithm presented here is largely based on the *expert model *(XM) compression model [27]. XM has been proved to be superior to other existing compression models thus giving the best estimate of the information content of sequences. In addition, its speed allows it to be applied to long sequences. Importantly, the expert model allows the compression of a sequence on the background of another, and can show references to the areas where better compression is achieved. These references make up the local alignment of the two sequences.

XM is a predictive model which can be used for compression of genomic sequences as well as to measure the information content of a sequence. It compresses each symbol of a sequence *X *by forming the probability distribution for the symbol based on the information from all symbols seen previously. The actual symbol is then encoded with respect to the probability distribution. The information content of the symbol is the theoretical length of the encoding of the symbol: ℐ(*x_i_*) = -*log*_2_*Pr*(*x_i_*).

In order to form the probability distribution of a symbol, the algorithm maintains a set of *experts*, whose predictions of the symbol are combined into a single probability distribution. An expert is any model that can potentially provide a reasonable probability distribution for the symbol. With the availability of a reference sequence, the sender and the receiver can recruit experts that base their predictions on the reference sequence. Expert opinions about the symbol are blended to give a combined prediction for the symbol. The reliability of an expert is evaluated from its past predictions. A reliable expert is given a high weight in the combination while an unreliable one has little influence on the prediction or may be even ignored.

### Type of experts

An expert can be anything that provides a reasonably good probability distribution for the symbol at a position in the sequence. A simple example is the order *m Markov expert *which uses a Markov model learnt from the statistics of all previous symbols to give the probability of the symbol in the context of *m *preceding symbols. Initially, the Markov expert does not have any prior knowledge of the sequence and thus gives a uniform distribution to a symbol. As the encoding proceeds, the Markov expert gives the probability that a nucleotide appears in the next position as the frequency of its occurring previously. Essentially, the Markov expert provides the background statistical distribution of nucleotides over the sequence. Different areas of a DNA sequence may have differing functions and thus may have different probability distributions. To account for this, another type of expert called the *local Markov expert *is employed. The local Markov expert estimates the probability of a symbol based on the statistics from the *local *history rather than the entire history of the sequence.

In order to align two sequences *X *and *Y *, the method attempts to compress sequence *X *(query sequence) on the background knowledge of sequence *Y *(reference sequence). It uses *align experts *each of which considers the region *x_n_..x_n+l _*in *X *to be aligned to a region *y_m_..y_m+l _*in *Y*. An align expert estimates the probability of symbol *x_n+i _*(*i *∈ 0..*l*) based on the corresponding symbol *y_m+i_*. It uses an adaptive code [29], learned from its correct predictions and its mistakes in the region, to predict *x_n+i_*. Two techniques are available for an align expert to learn its probability distribution for prediction. First, in the *counting *technique, each align expert keeps track of the number of correct and incorrect predictions, and gives the following probability to the letter at *y_m+i_*:

(1) $$Pr(x_{n+i}=y_{m+i})=p=\frac{r+1}{w+2}$$

where *w *is the window size over which the expert reviews its performance and *r *is the number of correct predictions the expert has made; the remaining probability, 1 - *p*, is distributed evenly to the other letters of the alphabet. Second, in the *substituting *technique, each align expert maintains a substitution matrix and give predictions according to the matrix.

If there is a mutation, the align expert gives a bad prediction at the position of the change, and its weight is decreased. However, subsequent correct predictions restore its influence in the combined prediction. On the other hand, when the homologous region ends, the align expert makes several mistaken predictions and its weight quickly decreases. When the weight of the expert drops to below a threshold, the expert is removed from the panel. This also happens when an insertion or a deletion occurs - the align expert is no longer able to make good predictions and is eventually excluded to make room for other align experts. Though each align expert can only utilise a gap free matching region for prediction, many align experts collectively can handle larger regions that contain deletions and insertions.

### Proposing align experts

When a symbol of query sequence *X *is encoded, there are 2|*Y ***| **possible align experts. This is too many to combine efficiently and anyway most are not *genuine *and thus would be ignored. To be efficient, the algorithm must use at most a small number of align experts at one time. The algorithm has a parameter *L*, which specifies the maximum number of align experts in use. When the expert panel size is less than *L*, the algorithm may recruit more potential align experts. Since the number of experts must be small to be efficient, it is desirable that the experts proposed are those most likely to be genuine experts.

A simple method to propose potential experts is by using a hash table. The hash table associates every position in the reference sequence with the hash key composed of *k *symbols *preceding *the position. It proposes experts that suggest the current symbol is homologous to the symbols in positions in *Y *having the same hash key. The choice of hash key size, *k*, and expert limit, *L*, is a trade-off between running time and compressibility, and hence alignment quality. Generally, a small *k *and a large *L *allow XMAligner to search for repeats more thoroughly and thus give better compression at the cost of more time.

Several techniques can be used to allow the hash table to propose align experts based on non-exact matching. There are two groups of nucleotides - purine (C and T) and pyrimidine (A and G). The biological properties of two nucleotides in a group are more similar than those from different groups. Therefore, substitutions changing nucleotides in a group (transitions) are more common than those that change the group (transversions). In order to permit mismatches in seeds, XMAligner provides an option to use the hash table on the alphabet {purine, pyrimidine}. Another technique is using gapped hash tables [30] which allow selecting align experts based on matching with errors at specified positions in the hash key.

Alternatively, a suffix tree or a suffix array can be used to propose align experts. These suffix structures allow selecting potential align experts based on the longest possible matching, especially for long sequences where random matches tend to be numerous. With a suffix structure, XMAligner can recruit up to *L *align experts from the *L *longest matches. Suffix structures can also operate on the alphabet {purine, pyrimidine}, but cannot suggest align experts from matches with errors.

### Combining expert predictions

Not only do experts adapt themselves based on the context of symbols they have seen, XMAligner also adaptively adjusts each expert's weight to reflect its accuracy in the given context. Good experts are assigned high weights. Even being nominated by the hash table, some align experts are just random matches and thus their predictions are not significantly better than the Markov experts. The algorithm must be able to exclude the by-random nominees to reduce noise and to be more efficient. Furthermore, a "genuine" align expert performs well only within a homologous region. Beyond this, it provides random predictions and thus should also be excluded. It is important that the algorithm is able to evaluate the goodness of each expert to assign a weight accordingly, and to exclude the expert when necessary.

The core part of the expert model is the evaluation and combination of expert predictions. Suppose at position *n *on the query sequence *X*, a panel of experts *E *is available to the compressor. Expert *θ_e _*gives the probability *Pr*(*x_n_*|*θ_e_*, *x*_1..*n*-1_) of symbol *x_n _*based on its observations of the preceding *n *- 1 symbols. The expert is assigned a weight $w_{\theta_{e}}$ which reflects its reliability. The expert model performs a linear blending of experts' predictions to give the probability distribution of the symbol *x_n_*:

(2) $$Pr(x_{n}|x_{1..n-1})=\sum_{\theta_{e}\in E}w_{\theta_{e}}Pr(x_{n}|\theta_{e},x_{1..n-1})$$

in which the sum of all weights is equal to 1:

(3) $$\sum_{\theta_{e}\in E}w_{\theta_{e}}=1$$

A sensible way to combine experts' predictions is based on Bayesian model averaging which sets an expert's weight to its posterior probability after encoding the previous *n *- 1 symbols.

(4) $$w_{\theta_{e}}=Pr(\theta_{e}|x_{1..n-1})$$

As has been shown in [31], this posterior probability of *θ_e _*is proportional to the product of its predictions of the *n *- 1 symbols. Therefore

(5) $$w_{\theta_{e}}\propto \prod_{i=1}^{n-1}Pr(x_{i}|\theta_{e},x_{1..i-1})$$

Taking the negative log of the two sides in Equation 5 gives

(6) $$-log_{2}(w_{\theta_{e}})\sim -\sum_{i=1}^{n-1}log_{2}Pr(x_{i}|\theta_{e},x_{1..i-1})$$

In other words, the negative logarithm of $w_{\theta_{e}}$ varies linearly with the length of the encoded subsequence *x*_1..*n*-1 _by expert *θ_e_*. To evaluate experts on a *recent *history of size *h*, only the message length of encoding symbols *x*_*n*-*h..n*-1 _is used to determine the weights of experts. The final formula of $w_{\theta_{e}}$ is

(7) $$w_{\theta_{e}}\propto 2^{-msgLen(x_{n-h..n-1}|\theta_{e})}$$

If a symbol is part of a homologous region, the align expert of that region must predict significantly better than a Markov expert. We therefore define a *listen threshold*, *T*, to determine the inclusion of an align expert. An align expert is considered reliable if the length of its encoding of the last *h *symbols is smaller that of the Markov experts by *T *bits. An align expert is expected to be involved in prediction of a homologous region. Beyond the region, its predictions becomes random and therefore its performance gets worse. If the align expert performance falls below the threshold, the expert is discarded to make way for others.

### Identifying similar regions

The main idea behind our alignment algorithm is that if two sequences are related, one will tell something new and useful about the other, that would not be known otherwise. If a region *R_x _*in the query sequence *X *has some biological relationship with some region *R_y _*in the reference sequence *Y *, the similarity between *R_x _*and *R_y _*should be better than random. The align expert based on *R_y _*should perform better on *R_x _*than the Markov experts whose predictions are based purely on the general statistics of sequence *X*. We therefore consider a region conserved if there is an align expert that predicts significantly better than the Markov experts in the region due to the shared information between the region and a related region in the reference sequence. The amount of shared information, measured in bits, indicates the similarity of the two regions. The more information shared, the more similar they are. Such a region is called a *High-scoring Segment Pair *(HSP).

The method identifies HSPs by considering high performing align experts. Each align expert is typically proposed by the hash table at some point in the query sequence during the compression process. It takes part in the compression until being discarded from the expert panel. The align expert assumes that the region it predicts is related to a region in the reference sequence, and bases its prediction on the assumption. The two regions form an HSP; the score is determined by the difference between the performance of the align expert and the Markov experts.

This sub-section shows that the alignment score of an HSP [32] is in fact the mutual information content of the pair. Consider an align expert that aligns nucleotide *x_i _*in *X *to nucleotide *y_j _*in *Y*. The alignment score is specified by the logarithm of the odds ratio of a model *H *which assumes the two nucleotides are homologous, and a model *R *assuming they are random:

(8) $$S(x_{i},y_{j})=log_{2}\frac{Pr(x_{i},y_{j}|H)}{Pr(x_{i},y_{j}|R)}$$

Since model *R *assumes that the occurrence of *x_i _*in *X *and *y_j _*in *Y *are independent, the denominator of the right hand side can be expressed as *Pr*(*x_i_*, *y_j _*|*R*) = *Pr*(*x_i_*)*Pr*(*y_j _*). On the other hand, model *H *considers symbol *x_i _*to be related to symbol *y_j _*and hence *Pr*(*x_i_*, *y_j _*|*H*) = *Pr*(*x_i_*|*y_j _*, *H*)*Pr*(*y_j _*) by Bayes's theorem. Therefore,

(9) $$\begin{array}{c} S(x_{i},y_{j})=log_{2}\frac{Pr(x_{i}|y_{j},H)Pr(y_{j})}{Pr(x_{i})Pr(y_{j})}\\ =log_{2}Pr(x_{i}|y_{j},H)-log_{2}Pr(x_{i}) \end{array}$$

*Pr*(*x_i_*|*y_j _*, *H*) is the probability of symbol *x_i _*estimated by the align expert upon observing *y_j _*while *Pr*(*x_i_*) is the probability of *x_i _*estimated by the Markov experts. *S*(*x_i_*, *y_j _*) thus, is the mutual information of the two symbols. The alignment score of an HSP is the sum of alignment scores of all symbols in the regions. If the HSP is from two regions starting at *x_n _*and *y_m _*respectively and is *l *symbols long, its alignment score is

(10) $$S\left(x_{n},y_{m},l\right)=\sum_{i=0}^{l-1}-log_{2}Pr(x_{n+i})-\sum_{i=0}^{l-1}-log_{2}Pr(x_{n+i}|y_{m+i},H)$$

The two terms are the lengths of the compressed messages of the region *x*_*n..n*+*l*-1 _by the Markov experts, and by the align expert, respectively. In other words, the alignment score of an HSP is the mutual information content of the two regions.

An HSP is considered a homologue if its alignment score is greater than a fraction of the information content of the region from the query sequence. Specifically, XMAligner has a parameter *homology ratio threshold r*, and selects HSPs having alignment scores

(11) $$S(x_{n},y_{m},l)\;>r\sum_{i=0}^{l-1}-log_{2}Pr(x_{n+i})$$

as the local alignment.

Once all the HSPs have been selected, overlapping HSPs and HSPs having distances less than a certain threshold are chained together to form larger regions. More specifically, two HSPs $\left(x_{m_{1}},y_{n_{1}},l_{1}\right)$ and $\left(x_{m_{2}},y_{n_{2}},l_{2}\right)$ where *m*_1 _**<***m*_2 _are considered close if the distances between the end of HSP $\left(x_{m_{1}},y_{n_{1}},l_{1}\right)$ and the beginning of HSP $\left(x_{m_{2}},y_{n_{2}},l_{2}\right)$ in both sequences are less than a predefined gap. The alignment score of a chain is the sum of the alignment scores of all HSPs involved. The alignment algorithm is formally described in Algorithm 1.

**Algorithm 1 **Expert Model Alignment Algorithm

   XMAligner(Sequence X, Y)

   param L: limit on size of the expert panel *E*

   param k: size of the hash key

   param r: the ratio threshold to determine statistically significant HSPs.

   param h: size of the window to evaluate experts

   param T: threshold to discard align experts

   Use the hash table to index every position of the reference sequence

   *E ***← **empty set

   **for ***n ***← **1 to |*X*| **do**

      **while **|*E*| **<***L ***do**

         **if **expert *θ_e _*which matches *y_m _*to *x_n _*is proposed **then**

            add *θ_e _*into *E*

            set *Start_X _*(*θ_e_*) **← ***n *{The starting point of expert *θ_e _*in query sequence *X*}

            set *Start_Y _*(*θ_e_*) **← ***m *{The starting point of expert *θ_e _*in reference sequence *Y *}

         **else**

            break {No expert is proposed}

      **end if**

      **end while**

      set $Pr(x_{n})\leftarrow \sum_{\theta_{e}\in E}w_{\theta_{e}}Pr(x_{i}|\theta_{e})$ where $w_{\theta_{e}}=2^{-msgLen\;(x_{n-h+1..n}}{{}^{|}}^{\theta_{e})}$

      *msgLen*(*x_n_*) **← **-*log*_2_*Pr*(*x_n_*)

      **for all ***θ_e _*∈ *E ***do**

         *msgLen*(*x_n_|θ_e_*) = -*log*_2_*Pr*(*x_n_|θ_e_*))

         update *θ_e_*

         **if ***msgLen*(*x_n-h_..x_n_|θ_e_*) > *msgLen*(*x_n-h_..x_n_|θ_Markov _*) - *T ***then**

            remove *θ_e _*from *E*

            set *l ***← ***n *- *Start_X _*(*θ_e_*)

            form an HSP that matches $x_{Start_{X}(\theta_{e}),\;l}\;\text{with}\;y_{Start_{Y}(\theta_{e}),\;l}$.

            set score $S\left(H\right)\leftarrow \sum_{i-0}^{l-1}-log_{2}Pr(x_{n-i}|\theta_{Markov})-\sum_{i=0}^{l-1}-log_{2}Pr(x_{n-i}|\theta_{e})$

            if $S(H)>r\sum_{i=0}^{l-1}-log_{2}Pr(x_{n-i}|\theta_{Markov})$**then**

               Add the HSP to a list

            **end if**

         **end if**

      **end for**

   **end for**

   chain sufficiently close HSPs together

## Results

We ran experiments to compare the performance of XMAligner to several common genomic alignment algorithms. The criteria for selecting these algorithms was that (i) they can align long sequences, and (ii) they are available to install on a workstation. The alignment algorithms selected for comparison included Dialign [9], Chaos [14], Sim4 [7], Blastz [13] and Nucmer and Promer in the MUM-mer package [12]. Experiments were run on a work station equipped with an Intel dual core 2.66 Ghz CPU with 8 GB of memory. The machine ran Linux Ubuntu 9.04.

We consider the use of genomic alignment tools in the context of identifying interesting regions in the genomes which in many cases are related to homologous regions [33]. We therefore evaluated the performance of each algorithm based on its ability to detect homologues. In statistics, *sensitivity *is defined as $Sn=\frac{TP}{TP+FN}$ and *precision *is defined as $Sp=\frac{TP}{TP+FP}$, where *TP *is the number of true positives, *FP *is the number of false positives, and *FN *is the number of false negatives. What constitutes a true positive etc. depends on what question is asked. The literature takes two approaches: (1) Does the method correctly identify that a segment of the query sequence is related to some segment or segments of the reference sequence? (2) Does the method correctly identify the exact base in the reference sequence to which a base within a segment of the query sequence corresponds? Clearly, both questions have their place.

We consider true positives (*TP*) to be the number of homologous nucleotides that are correctly predicted as homologous (i.e., are aligned with some nucleotides in the reference sequence by the alignment tool), true negatives (*TN*) to be the number of non-homologous nucleotides that are correctly predicted as non-homologous, false positives (*FP*) to be the number of non-homologous nucleotides that are incorrectly predicted to be homologous, and false negatives (*FN*) as the number of homologous nucleotides that are incorrectly predicted to be non-homologous. This definition corresponds to asking question (1) above.

In statistics, specificity is traditionally defined as $\frac{TN}{TN+FP}$. However, for alignments there are generally many fewer homologous regions, and thus homologous nucleotides, than non-homologous regions. So *TN *tends to be much higher than *FP *, making the traditional formula uninformative. Consequently, the formula for *Sp *is typically used for *specificity *in alignment applications [34]. These same definitions - *Sn *and *Sp *with respect to question 1) - have been used to compare tools for coding regions identification [14,33]. Some work [13,35] addressing question (2) above, define a quantity called *alignment coverage*; this happens to be equivalent to *Sn *for question (1)! Although this quantity does not necessarily account for the exact matching of nucleotides, it is expected to be "highly correlated with alignment sensitivity" for question (2) [[36], p. 764]. In words, the definitions used herein are: *sensitivity *(*Sn*) is the fraction of homologous nucleotide sites covered by the alignments predicted; and *specificity *(*Sp*) is the fraction of homologous nucleotide sites predicted that are true homologues. Where possible, the receiver operator characteristics (ROC) curve, plotting sensitivity against specificity, for each algorithm is presented.

### Simulated data

An evaluation of an alignment tool compares the homologues predicted by the tool against "true" homologues. True homologues in genomes, however, are not always reliable as they are often located by automated tools or by subjective prediction by human experts. Some alignment benchmarks based on real data such as BAliBASE [37] and Jareborg [38] were designed based on manually curated alignments and structure protein information. These benchmarks are therefore only restricted to short sequences, and to homologues from protein coding regions. Since some conserved regions are not necessarily protein coding, these benchmark data sets may cause alignment tools to report "wrong" false positives. Simulated data benchmarks, such as those proposed in [35] and [39], are guaranteed to provide the true answers to alignment. The use of simulated data sets also allows exploring the entire spectrum of the problem space. These benchmarks, however, contain only short homologous sequences (1 kb - 10 kb) and are only suitable for global alignment tools. They thus do not meet our goal of evaluating genome alignment tools.

We first experimented using simulated data. We generated our artificial genome benchmark data set in which homologous regions are scattered around the genomes in a random order. These homologous regions were taken from the alignment benchmark in [39] for which the generation was inspired by non-coding regions from the *Drosophila *genomes. We selected ten alignments at random from their 10000 alignments. Each alignment contains homologous sequences that were generated based on homologous non-coding regions of five species *Drosophila melanogaster*, *Drosophila simulans*, *Drosophila yakuba*, *Drosophila ananassae *and *Drosophila mojavensis*. Each sequence is 1000 bases long. We generated five *unrelated *simulated genomes of length 100 kb, and inserted the ten homologous sequences of each species into a simulated genome at random positions. The generation resulted in five simulated genomes, each of which contains ten homologous regions.

We performed local pairwise alignment of the simulated genome containing *D. melanogaster *homologous sequences against each of the other four genomes. The object of the alignment was to locate the homologous sites from each genome. Sites resulting from insertions were not considered homologous. The data set consists of four pairs of simulated genomes, namely, *D. melanogaster *- *D. simulans*, *D. melanogaster *- *D. yakuba*, *D. melanogaster *- *D. ananassae *and *D. melanogaster *- *D. mojavensis *in order of increasing genetic distance. In order to investigate different statistical distributions, we generated two sets with different statistical properties from these four pairs. In the first set, unrelated regions were generated from a uniform distribution (uniform set). In the second set, unrelated regions were generated from a statistically biased distribution in which the frequencies of A, C, G and T are 40%, 10%, 10% and 40% respectively (biased set). In total, our benchmark contained eight pairs of simulated genomes.

The programs XMAligner, Dialign [9], Chaos [14], Nucmer [12] and Blastz [13] were applied to each pair of sequences. Promer and Sim4 were not included because they either perform alignment at the amino acid level, or rely on finding exon boundaries, whereas the data generated exhibit substitutions at the nucleotide level. Furthermore, the simulated homologous regions are not actual coding regions and hence cannot sensibly be translated to protein. For each program used, we made an effort to choose the best possible parameters for a specific pair of sequences. We then varied one parameter to get different values of sensitivity and specificity of each algorithm. In particular, for Chaos, we varied the *score cut-off *(co) and set the *word length *(wl) to 10; for Dialign, we varied the *threshold *(thr); for Nucmer, we varied *min cluster *(c) and set *minmatch *(l) to 14 and *maxgap *(g) to 120; for Blastz, we varied *scoring threshold *(hspthresh); for XMAligner, we varied *homology ratio threshold *(r) and set the hash key size to 10. All other parameters were set to their default values for DNA alignment.

**Figure 2:**
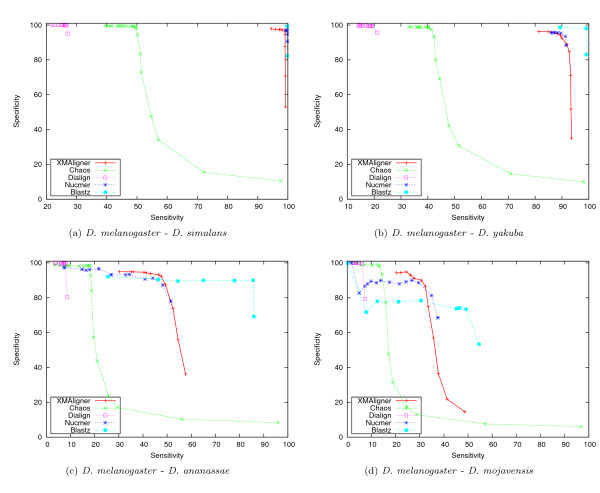
**Comparative performance on the uniform set**.

**Figure 3:**
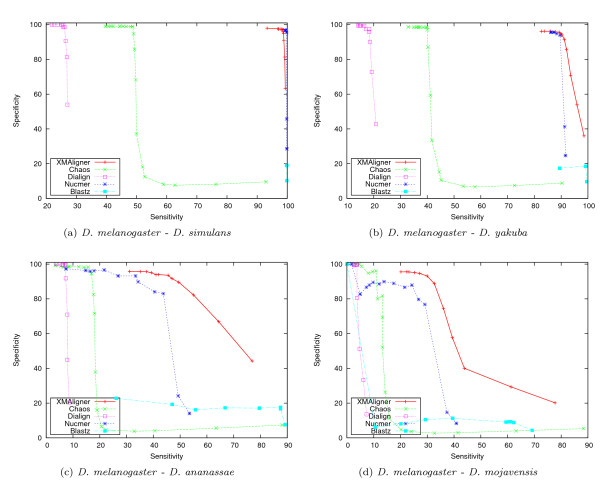
**Comparative performance on the biased set**.

Figure 2 and Figure 3 show the performance of these algorithms on the four sequence pairs in the uniform and biased sets, respectively. Generally, the performance of the algorithms deteriorated with increasing genetic distances. In all cases, XMAligner, Nucmer and Blastz were clearly superior to Chaos and Dialign. On the uniform set, XMAligner performed comparably to Nucmer. Blastz was the most sensitive among the five programs. However, it was less specific than XMA-ligner and Nucmer on distantly related sequence pairs such as *D. melanogaster *- *D. ananassae *and *D. melanogaster *- *D. mojavensis*. On the biased set, XMAligner outperformed both Blastz and Nucmer, especially on distantly related sequences. In such biased data, spurious matches occur more often than in more uniformly distributed data. As a result, Blastz, Chaos and Nucmer were misled by the bias of the data. On the other hand, XMAligner examines the information content of every nucleotide. In a low information region, the information content of a non-homologous nucleotide is calculated accordingly and thus spurious matches reported are minimised.

**Figure 4:**
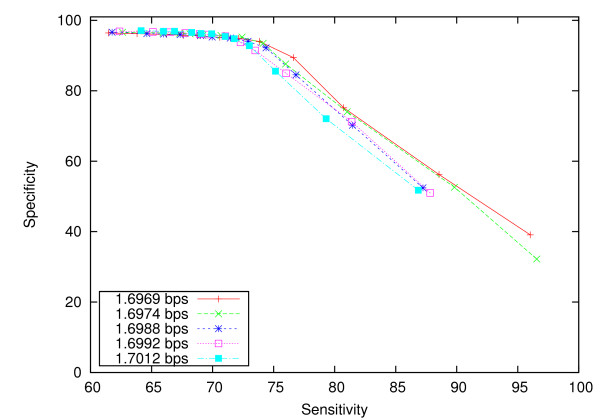
**Relationship between compressibility and alignment performance**.

We performed an experiment to verify the proposition that the best alignment of two sequences leads to the best compression of a sequence on the background of the other. The experiment was performed on the four biased genome pairs. We first varied the parameters of the compression model, namely the hash table key size, the context length and the expert panel limit, so that different compression results could be obtained. The compression performance of each set of parameters is measured by the average compression of the simulated *D. melanogaster *genome in each pair. For each set of parameters, we varied the homology ratio threshold to obtain different sensitivity and specificity values. The ROC curve for each set of model parameters is displayed in Figure 4, and is labelled by the compression result, in bit per symbol. The two configurations that produced the best compression results, 1.6969 bps and 1.6974 bps, also gave the best alignment performance. On the other hand, the configurations that produced the worst compression results (1.6992 bps and 1.7012 bps) were inferior to other configurations set up in the experiment.

### Human-Mouse data set

We also performed experiments on real data. We used the Jareborg data set [38] which contains 42 annotated pairs of genomic sequences from the mouse and human genomes. These sequences vary in length between 6 kilobases to 220 kilobases, with an average length of 38 kilobases. They contain 77 verified exon pairs. As exons are under stronger selective pressure, they tend to be more conserved than non-coding regions. The performance of an alignment algorithm is often evaluated by its ability to detect exons. Indeed, the data set was used to evaluate alignment algorithms in several previous studies [14,33].

**Figure 5:**
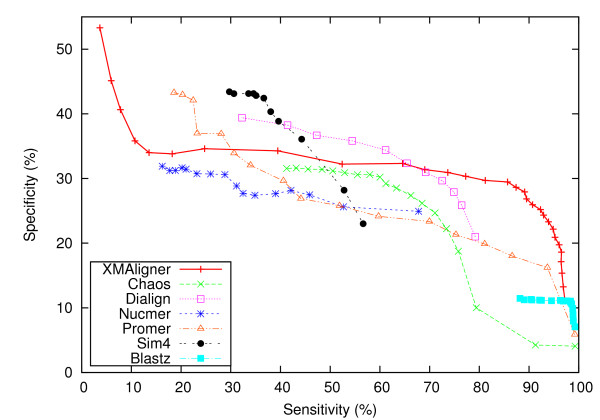
**Performance comparison on Human-Mouse data set**.

For a pair from the data set, we applied each algorithm to align the mouse sequence against the human sequence, and compared the HSPs detected in the mouse sequence to the annotated mouse exons. The parameters for XMAligner, Chaos, Dialign, Nucmer and Blastz were the same as in the previous experiment. For Promer, we varied *min cluster *(c) and set *minmatch *(l) to 6 and *maxgap *to 30; for Sim4, we varied HSP threshold (C) and set word size (W) to 10. The sensitivity versus specificity ROC curves for these algorithms are plotted in Figure 5. In general, XMAligner was the most sensitive among the algorithms in the experiment. In particular, it outperformed Blastz Chaos and Nucmer which also align sequences at the DNA level. Other methods, which either translate potential exons to proteins and perform alignment at the protein level (Promer and Dialign) or use a built-in exon boundary detection mechanism, are more specific.

### Malaria parasite genomes

We used XMAligner to align the genomes of five *Plasmodium *species, namely *P. falciparum, P. knowlesi, P. vivax, P. gallinaceum *and *P. yoelii*. The genome sequences and their annotations were obtained from PlasmoDB release 6.2 [40]. Of the five species, *P. falciparum *and *P. vivax *are malaria parasites on human while *P. knowlesi *and *P. yoelii *cause malaria in monkey and rodent respectively. *P. gallinaceum *is a bird malaria parasite. The nucleotide compositions in these genomes are very different. The AT content in the genome of *P. falciparum *is as high as 80% genome-wide, even 90% in introns and intergenic regions, while the AT content in the *P. vivax *genome is just 57.60%.

The genomes of *Plasmodium *species exhibit an extremely difficult example of sequence alignment. The highly skewed distributions of genomes of species such as *P. falciparum*, especially in non-coding regions, may lead to the return of spurious matches. Furthermore, in different stages of their life-cycle, *Plasmodium *species interact with the mosquito vector and the vertebrate host. The strong evolutionary pressure from these interactions has resulted in different codon preferences among the genomes of *Plasmodium *species. Indeed, the AT content of coding regions of *P. falciparum *is as high as 76% while the AT content of coding regions of another human malaria parasite, *P. vivax *is only 53%, although the two species have similar metabolic pathways and their proteins share a high level of identity [41].

We aligned each of the *P. falciparum *and *P*. *knowlesi *genomes against each of four other genomes and against the concatenation of these four genomes. The similar regions detected during alignment were compared with the exon annotation. We compared XMAligner with Blastz [13], Promer and Nucmer [12], which are the only three among the chosen programs able to align such long sequences. Blastz and Nucmer align the sequences at the nucleotide level while Promer translates potential exons to protein and aligns at the protein level. Promer is generally used when the sequences are relatively divergent, which Nucmer cannot handle. We varied the parameters *scoring threshold *(hspthresh) of Blastz, *minimum cluster *(c) of Nucmer and Promer, and *homology ratio threshold *(r) of XMAligner to get several different values of sensitivity. Other options are presented in Table 1 and Table 2.

**Table 1:** Sensitivity and specificity of exon detection from the P. falciparum genome

Method & params	*P.f */*P.g*		*P.f */*P.k*		*P.f */*P.v*		*P.f */*P.y*		*P.k */All		Total Time (Mins)
		
	Sen. (%)	Spe. (%)	Sen. (%)	Spe. (%)	Sen. (%)	Spe. (%)	Sen. (%)	Spe. (%)	Sen. (%)	Spe. (%)	
XMAligner -*hashSize = 20 *-*binaryHash = true *-*limit = 500*											
r = 0.15	76.44	75.94	57.61	83.53	55.94	86.21	75.81	81.49	80.12	79.71	451.62
r = 0.25	51.83	86.50	42.03	90.81	39.60	91.65	52.22	89.45	59.73	88.63	441.40
r = 0.35	35.37	93.55	31.13	94.54	28.76	94.58	36.11	93.92	44.08	93.31	439.53
Promer *-l 6 -g 30*											
c = 10	78.43	50.48	66.88	51.13	62.66	51.61	80.37	51.80	87.55	52.85	327.21
c = 20	46.23	78.72	43.15	89.13	39.76	92.35	48.98	83.14	54.16	79.72	33.39
c = 40	34.38	86.36	29.83	95.92	27.13	97.32	32.92	90.01	31.14	87.89	28.23
Nucmer *-l 14 -g 160*											
c = 40	18.94	73.82	6.64	52.99	3.61	41.80	17.54	74.50	22.12	72.79	17.76
c = 65	14.63	94.65	3.71	88.44	1.71	76.98	12.86	95.93	16.01	94.42	7.89
c = 90	11.69	97.10	2.22	87.52	0.86	73.31	10.08	97.33	12.20	96.65	6.66
Blastz -*notransition *-*step = 20 *-*nogapped*											
t = 3000	34.24	75.35	19.26	69.03	13.74	73.91	30.96	82.19	44.97	75.65	34.57
t = 5000	28.16	95.56	15.51	93.58	11.43	94.55	25.82	96.43	37.89	94.78	31.23
t = 7000	24.73	96.88	14.05	96.02	10.69	95.88	23.43	97.03	33.31	96.35	30.89

**Table 2:** Sensitivity and specificity of exon detection from the P. knowlesi genome

Method & params	*P.k */*P.f*		*P.k */*P.g*		*P.k */*P.v*		*P.k */*P.y*		*P.k */All		Total time (Mins)
		
	Sen. (%)	Spe. (%)	Sen. (%)	Spe. (%)	Sen. (%)	Spe. (%)	Sen. (%)	Spe. (%)	Sen. (%)	Spe. (%)	
XMAligner -*hashSize = 20 *-*binaryHash = true*-*limit = 500*											
r = 0.15	91.73	51.62	89.04	52.50	98.23	51.48	90.74	52.68	98.77	50.57	470.40
r = 0.25	61.82	63.02	52.38	64.83	93.49	57.09	59.61	66.42	93.30	57.06	450.56
r = 0.35	42.12	82.86	34.05	84.74	90.01	62.06	40.78	85.89	88.64	63.87	446.37
Promer *-l 6 -g 30*											
c = 10	60.32	60.80	47.52	58.10	94.49	54.89	57.67	63.35	94.89	54.28	109.48
c = 20	45.55	90.53	37.44	91.90	92.11	67.16	43.62	91.82	92.07	67.64	41.37
c = 40	32.40	95.28	28.60	96.46	85.67	79.62	30.40	95.50	84.60	80.12	33.99
Nucmer *-l 14 -g 160*											
c = 40	6.31	69.61	6.36	74.28	71.59	60.98	6.31	75.44	70.84	61.14	14.05
c = 65	3.38	75.40	3.51	81.34	64.98	63.26	3.33	77.86	63.71	63.73	9.96
c = 90	1.80	74.80	1.93	83.34	58.74	65.22	1.82	80.71	57.16	65.76	8.26
Blastz -*notransition *-*step = 20 *-*nogapped*											
t = 3000	17.12	75.97	15.21	77.42	74.92	72.89	16.87	79.64	75.94	72.01	24.04
t = 5000	16.24	83.96	14.62	82.64	74.29	77.23	16.10	86.48	75.06	76.59	21.04
t = 7000	15.55	87.05	14.20	85.75	73.40	81.31	15.43	89.03	73.97	80.70	20.57

The alignment of one genome against another by XMAligner took about 40 minutes. To get high sensitivity, we performed alignment in both forward and reverse directions, and then combined both alignments. The total time for alignment of a pair of sequences therefore was about 80 minutes. The running time of Promer was shorter, about 4 to 5 minutes for alignment one genome against another, and 20 minutes to align one genome against the four other genomes. Nucmer is even faster, it needed only one minute for pairwise alignment and four minutes for aligning one against four genomes.

The sensitivity and specificity of exon detection of the three programs on the genomes of *P. falciparum *and *P. knowlesi *are shown in Table 1 and Table 2 respectively. A column with the header X/Y shows the performance of aligning the genome of × against the genome of Y and a column with header X/ALL shows the performance of aligning the genome of × against the other four genomes.

Nucmer performed poorly on most cases, with the exception of aligning the *P. knowlesi *genome against *P. vivax*, these being closely related. In the alignment of distantly related genomes, Nucmer obtained a sensitivity of no more than 20% in most cases. Promer performed significantly better than Nucmer on the data, although the matching techniques of the two algorithms are similar, except that Promer performs alignment at the protein level while Nucmer aligns at the nucleotide level. Blastz performed better than Nucmer, but was inferior to Promer on aligning these sequences.

Although XMAligner aligns sequences at the nucleotide level (i.e., it does not take exons and protein into account), it showed a much higher level of both sensitivity and specificity than Promer in the alignment of most pairs. The only exception is the closely related pair *P. knowlesi *and *P. vivax*, where XMAligner was more sensitive but less specific. With such a close relationship, many regions other than exons also tend to be conserved. While Promer translates DNA to proteins for alignment, the annotation of just codons is clearly advantageous to Promer's specificity.

## Visualisation of alignment

We have incorporated the output of XMAligner into InfoV toolkit [42] for visualisation. When aligning a sequence *X *against a sequence *Y *, XMAligner outputs the sequence of information content of *X *and the sequence of the conditional content of *X *given *Y *, along with a list of HSPs. The toolkit can read these information sequences, manipulate and display them. The annotation of the sequences can also be visualised by the toolkit.

**Figure 6:**
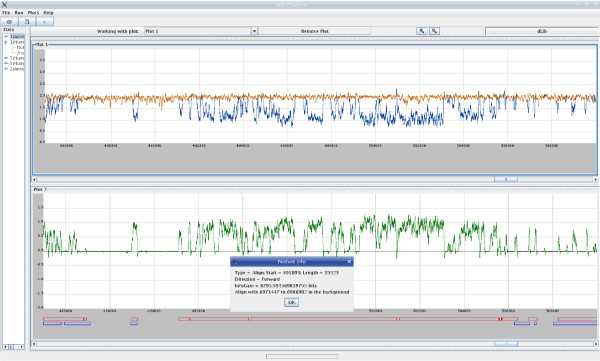
**Visualisation of the alignment of the P. vivax contig ctg6843 against the P. falciparum genome**.

In an earlier publication [43], we performed an alignment experiment using XMAligner and InfoV toolkit. We downloaded the *P. vivax *and *P. falciparum *genomes from PlasmoDB version 5.4. We applied XMAligner to align contig ctg6843 from the *P. vivax *genome against the genomes of *P. falciparum*. The information content sequence of the contig and the conditional information content sequence of the contig given the *P. falciparum *genome were generated by XMAligner. The information content sequences were loaded into InfoV for viewing. The visualisation of these information sequences and the alignment is shown in Figure 6. The top canvas plots the two information sequences. The mutual information, obtained by taking the difference of the two information sequences, is plotted in the bottom canvas.

InfoV is able to display the annotations of a sequence and the HSPs from an alignment. The two rows of red and blue boxes near the bottom of the viewer in Figure 6 display the HSPs from the alignment and the exon annotation of contig ctg6843 from PlasmoDB version 5.4. When a box is clicked, a pop up windows shows the relevant information of the HSP or of the annotation. Users can zoom in and out to view particular areas of interest. Figure 6 shows the view from position 485000 to 510000 of the contig.

During our experiment, we noticed a cluster of HSPs which paired regions in contig ctg6843 to some annotated coding regions in the genome of *P. falciparum*. These regions showed a high level of similarity but was not annotated in PlasmoDB 5.4 version. The cluster of these region starts at position 491038 in the ctg6843, and is about 15000 bases long. Its counterpart from the *P. falciparum *genome starts at position 6971447. We tracked down and found that this area in the *P. falciparum *genome is a cluster of three genes *MAL7P1.203*, *MAL7P1.320 *and *MAL7P1.204*. The information of the alignment of an HSP is shown in Figure 6. The area was thought to be a synteny region conserved across malaria species, and contain some genes [43]. A later version of PlasmoDB (release 6.2 [40]) verified this finding and annotated the area as gene *PVX 081792 *in the *P. vivax *genome.

## Discussion

Most genomic alignment methods have four major components: (i) an indexing technique for locating seeds, (ii) a method for extending seeds, (iii) a method for assigning score to each local alignment, and (iv) a method to evaluate the significance of an alignment. XMAligner presents novel technique for (ii), (iii) and (iv) while it can use any existing methods for (i) from conventional alignment approaches for to propose align experts. Indeed, XMAligner provides option to use hash tables, gapped hash table, suffix trees and suffix arrays, on the standard alphabet (i.e., A, C, G and T) or on the {purine, pyrimidine} alphabet. Other techniques will be implemented in the near future. Most importantly, the suitability of each seeding technique can be measured by the compression objective function.

With reference to the traditional dynamic programming approach, an align expert proceeds diagonally. This is similar to gap-free extending seeds. However, there can be more than one align expert employed at any time. If there are gaps in a homologous region, some neighbouring expert(s) would be proposed. Though each align expert can suggest a gap-free HSP, the panel of experts in XMAligner can handle gaps implicitly. This also allows XMAligner not to make any assumptions about gap scores.

The matching scores in the traditional dynamic programming approach are calculated based on an information theory perspective [32]. Indeed, an entry in the common substitution matrices such as PAM [44] and BLOSUM [45] represents the logarithm of the ratio of the probabilities of two hypotheses: the pair is homologous and the pair is random. These scores are calculated based on some pre-aligned data or under some evolutionary assumptions. These substitution matrices are therefore not suitable for alignment of sequences that have different properties to the data used to construct the matrices, such as sequences of biased composition. A previous attempt has been made to construct substitution matrices for such sequences by collecting pre-aligned sequences with similar composition statistics [46]. However, the suitability of the collected data and the reliability of the pre-alignment are called into question. We argue that it is desirable to estimate these probabilities from the sequences at hand. This calculation better reflects the information content of each symbol of the sequences to be aligned. These scores can even be estimated if the sequences are sufficiently long [31].

Equation 11 shows that the mutual information of an HSP is in fact the traditional alignment score of the HSP which is also measured by the logarithm of the odds ratio of the probability that two symbols are related and the probability that they are independent. However, XMAligner adaptively estimates these probabilities based on the context of the pair of symbols. For example, in a low information region, the information content of a more frequent symbol is lower and its alignment score is computed accordingly. Unlike the "pairwise statistical significance" approach in [47] which locally selects a scoring scheme from a pre-computed set, our approach estimates the scoring scheme directly from data. This mechanism of XMAligner also differs from other methodologies in dealing with biased composition data; for example in [48] where the scoring scheme is derived from the standard substitution matrix by an heuristic transformation and in [49] which estimates the statistical significance E-value from data. Furthermore, each align expert also adaptively estimates mutation rates based on its observed data and keeps a separate scoring scheme. With the compression criterion, experts with good scoring scheme are retained while experts with unreasonable scoring scheme are discarded early. As a result, the new methodology performs better than traditional methods on statistically biased data, as demonstrated in the Results section.

XMAligner might find multiple segments in the reference sequence that are strongly related to a similar segment in the query sequence. The degree of relatedness is specified by the conditional information content of the segment given each related segment on the reference sequence. This can be used as a ranking to guide further investigation of such an identified segment.

Most existing alignment algorithms lack an objective function to indicate which parameters are the most suitable for the data. Objective functions are very important for applications like sequence alignment because biological data are so diverse. It is very hard to anticipate which parameter values capture the essence of the data and will give the best results, especially for data that are not well studied. The objective function provided by XMAligner naturally guides parameter estimation and improves alignment quality.

## Conclusions

This article presents XMAligner, a novel sequence alignment approach that matches long sequences at the information content level. It considers the information content of the nucleotide at each position during the alignment process. The information content is determined by examining the context of the nucleotide. Unlike traditional alignment algorithms, XMAligner reports aligned regions from two sequences if there is significant shared information between the two regions. The approach is shown to outperform the conventional character-matching approaches, especially for distantly related sequences and sequences with statistically biased composition. The method is able to align eukaryote genomes with only a modest hardware requirement. The output from XMAligner can be integrated into a visualisation tool to aid the analysis of sequences.

We argue that, since genomic sequences are meant to carry information, aligning in terms of information content is a better approach for genomic sequence alignment. Each nucleotide should be examined within its context. The approach is better suited than the conventional approaches which measure the alignment score of matching symbols entirely based on a fixed scoring scheme.

## Authors' contributions

MDC developed methods, performed experiments, analysed data and wrote the paper. MDC, TID and LA contributed to the mathematics. TID and LA supervised the work, and participated in discussions on algorithms, biology and statistics, and in the writing of the paper. All the authors read, edited and approved the final manuscript.
